# Endovascular management of carotid-cavernous fistulas: a 16-year retrospective analysis of multimodal treatment strategies and long-term clinical outcomes

**DOI:** 10.3389/fneur.2025.1625899

**Published:** 2025-08-26

**Authors:** Chingiz Nurimanov, Iroda Mammadinova, Karashash Menlibayeva, Yeldar Kydyrmoldin, Ramazan Duisengali, Diana Kerimbayeva, Serik Akshulakov, Yerbol Makhambetov

**Affiliations:** ^1^Department of Vascular and Functional Neurosurgery, National Center of Neurosurgery, Astana, Kazakhstan; ^2^Department of Population Health Sciences, Faculty of Life Sciences and Medicine, King’s College London, London, United Kingdom

**Keywords:** carotid-cavernous fistulas, endovascular treatment, transarterial approach, transvenous approach, long term results

## Abstract

**Background:**

Carotid-cavernous fistulas (CCFs) are abnormal connections between the carotid artery and/or its branches and the cavernous sinus, potentially resulting in significant complications, including visual impairment and neurological deficits. Timely diagnosis and appropriate therapeutic intervention are critical to minimizing the risk of adverse outcomes. Over the years, endovascular techniques have become the preferred method for treating CCFs, offering high success rates with fewer complications. This study aims to study the clinical and radiological outcomes of patients with CCFs treated with endovascular approaches, and to identify key prognostic factors associated with treatment efficacy and symptomatic resolution.

**Methods:**

A retrospective analysis was conducted of patients diagnosed with carotid-cavernous fistulas (CCFs) and treated at the National Center for Neurosurgery in Astana, Kazakhstan, between 2008 and 2024. A total of 71 patients underwent endovascular embolization using either transarterial or transvenous techniques. According to the Barrow classification, type A CCF were observed in 62 patients (87.3%), type D in 7 patients (9.9%), and types B and C in 1 patient each (1.4%). Collected data included patient demographics, type of embolic materials utilized, number of procedures performed, treatment approach, and corresponding radiological and clinical outcomes.

**Results:**

Among the 71 patients included in the study, 80.3% achieved complete clinical resolution, 15.5% demonstrated partial improvement, and 4.2% experienced no clinical benefit. The mean age was 35.7 ± 12.9 years, with 71.8% males. Clinical outcomes were significantly associated with complete occlusion following the initial intervention (*p* < 0.001), with 96% of these patients achieving full symptom resolution. Imaging follow-up was available in all patients, with complete occlusion confirmed in 65 cases (91.5%) on MRI at 6 months. Other variables, including distal internal carotid artery flow (*p* = 0.145), number of interventions (*p* = 0.838), treatment approach (*p* = 0.529), and type of embolic agent employed (*p* = 0.778), did not demonstrate a statistically significant association with clinical outcomes.

**Conclusion:**

Endovascular embolization is a safe and effective first-line treatment modality for CCFs. Both transarterial and transvenous approaches offer comparable rates of clinical success. Achieving complete occlusion is a critical determinant of favorable clinical outcomes and is associated with a reduced risk of treatment-related complications.

## 1 Introduction

Carotid-cavernous fistulas (CCFs) are abnormal vascular connections between the carotid artery and/or its branches and the cavernous sinus. These lesions are categorized according to their etiology (traumatic or spontaneous), hemodynamic profile (high-flow or low-flow), and anatomical features. The most widely adopted classification system divides CCFs into direct and indirect types, with further sub classification based on flow dynamics and underlying etiological factors (1, 2).

The clinical presentation of CCFs is often variable and nonspecific; however, ophthalmic manifestations are the most common, primarily due to abnormal venous drainage from the orbit into the cavernous sinus (3). Early and accurate diagnosis, followed by prompt intervention, is crucial to preventing irreversible visual impairment and potentially life-threatening complications. The principal therapeutic objective in the management of CCFs is the complete occlusion of the fistulous connection while preserving physiological blood flow through the internal carotid artery (ICA) (4).

Historically, surgical ligation of the common carotid artery (CCA) represented the standard of care (5). However, the paradigm shifted significantly following Serbinenko’s introduction of detachable latex balloons in 1974 (6), which marked the advent of endovascular techniques in the treatment of cerebrovascular lesions. Since then, advancements in endovascular technology have substantially broadened the range of therapeutic options and have established endovascular embolization as the gold standard in CCF management, owing to its high efficacy and favorable safety profile.

Current endovascular strategies include transarterial or transvenous embolization, employing a range of embolic agents such as detachable coils (7), cyanoacrylates (8), or covered stents (9), each demonstrating variable rates of occlusion success. Although open surgical and radiosurgical interventions remain available, they are generally reserved as second-line or adjunctive therapies (10).

This study presents our institutional experience in the management of CCFs using various endovascular techniques and embolic materials. The aim is to analyze the various treatment strategies employed, classified by etiology, hemodynamic characteristics, and anatomical features, with the emphasis on long-term clinical and radiological outcomes during follow-up.

## 2 Materials and methods

### 2.1 Demographic and clinical characteristics

**Figure 1:**
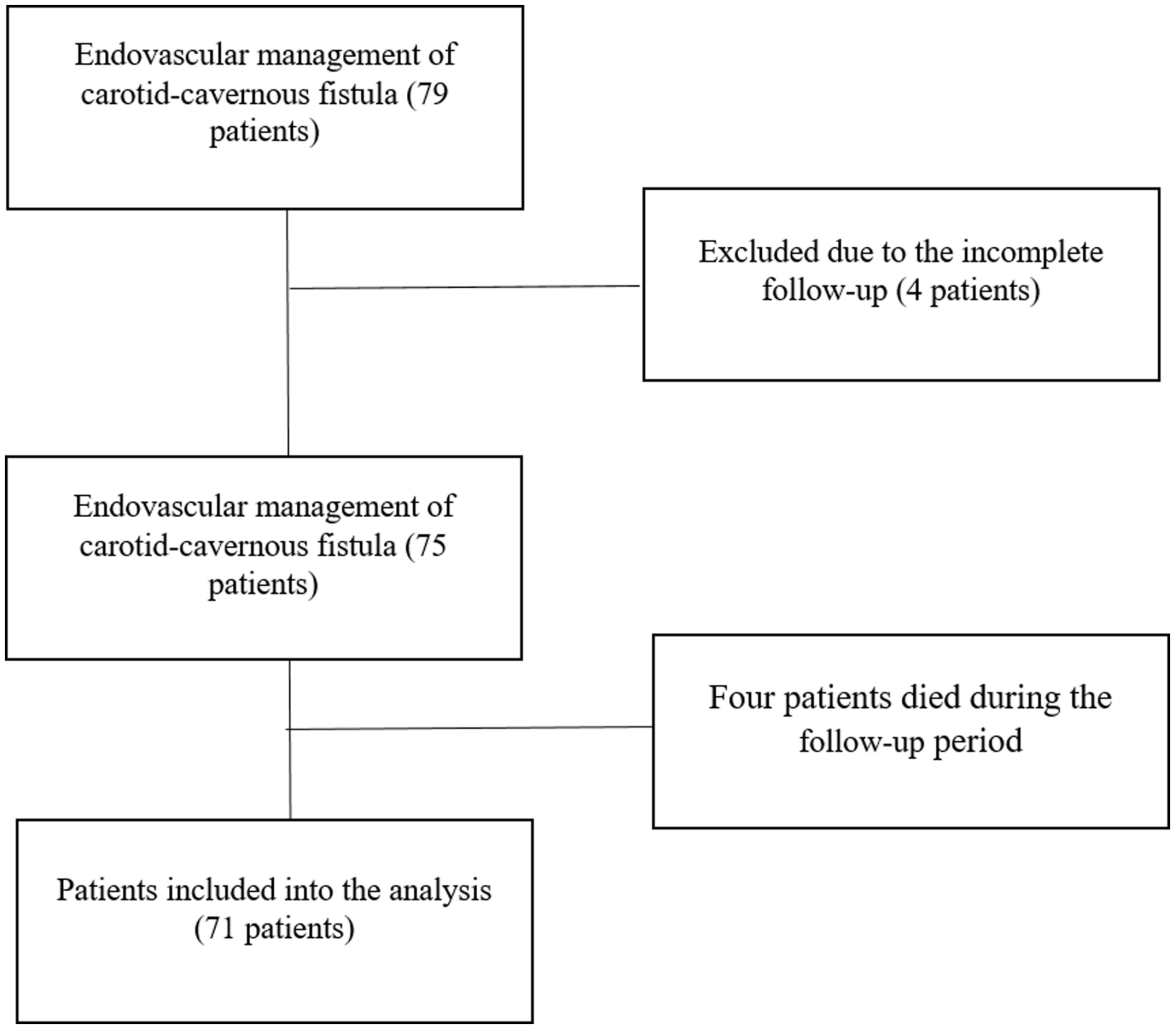
Patient inclusion and exclusion flow chart.

A retrospective review was conducted on all patients diagnosed with CCFs who underwent one or more endovascular treatment sessions at the National Center for Neurosurgery (Astana, Kazakhstan) between August 2008 and December 2024. A total of 79 patients who underwent embolization were initially identified for inclusion. Four patients were lost to follow-up due to unavailable follow-up MRI, and four had died by the time of data collection. Although the exact causes of death were not clearly defined, 3 of the 4 deceased patients were over the age of 60. Consequently, 71 patients were included in the final analysis (Figure 1).

Data collected for each patient included demographic variables (age and sex), type of embolic materials employed (coils, cyanoacrylate glue, stents, or combinations thereof), endovascular approach (transarterial or transvenous), clinical presentation, and perioperative and postoperative complications. Clinical presentation was categorized into four groups based on symptomatology:

*Ocular manifestations*: vision loss, ocular pain, glaucoma, chemosis, exophthalmos.

*Cavernous manifestations*: ophthalmoplegia, diplopia, ptosis, anisocoria.

*Cortical manifestations*: intracranial hemorrhage, seizures, focal neurological deficits.

*Petrosal manifestations*: tinnitus.

CCFs were diagnosed using magnetic resonance imaging (MRI) and digital subtraction angiography (DSA) with a biplane system (Artis Zee Biplane; Siemens, Erlangen, Germany). A multidisciplinary team of neuroradiologists and neurosurgeons reviewed the images and classified CCFs according to the Barrow classification:

Type A: Direct fistulas.

Types B, C, and D: indirect fistulas.

Additionally, fistulas were categorized based on etiology (traumatic or spontaneous) and hemodynamic properties (high-flow or low-flow), determined through angiographic evaluation. The updated classification system proposed by Thomas et al. was also applied, incorporating venous drainage patterns and their correlation with clinical symptoms, treatment planning, and outcomes. Additionally, contralateral cavernous sinus filling was assessed.

### 2.2 Treatment

All patients underwent endovascular treatment, which involved transarterial or transvenous embolization of the CCF or occlusion of the ICA. The choice of treatment approach was selected by the complexity of the fistula and the patient’s clinical presentation. Interventions were performed as either single-stage or multistep procedures.

**Figure 2:**
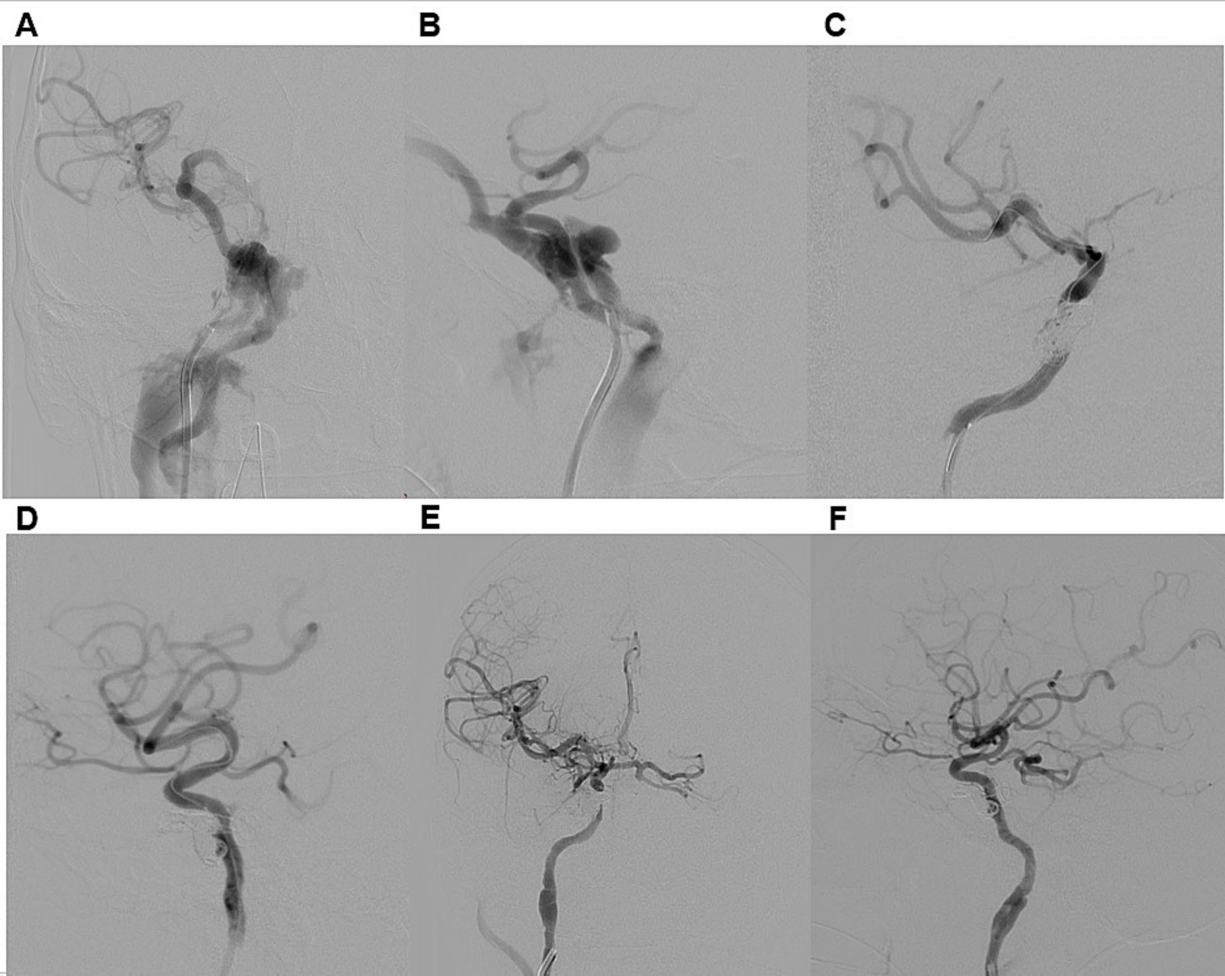
Illustrative case 1. A 34-year-old male presented with a six-month history of persistent pulsatile tinnitus, progressive proptosis, and conjunctival injection in the right eye, accompanied by visual impairment in the same eye after the motorcycle accident. DSA **(A,B)** confirmed a direct dural carotid-cavernous fistula on the right side, classified as Type A according to Barrow et al. and Type 5 according to the Thomas classification system. Venous drainage was retrograde through an enlarged right superior ophthalmic vein, the pterygoid venous plexus, and the right inferior petrosal sinus. Distal ICA flow was reduced, though both the anterior and posterior communicating artery were patent. The patient underwent transarterial embolization of CCF **(C,D)**. A 6F guiding catheter was introduced, and a HyperGlide 4 × 10 mm balloon catheter was positioned in the cavernous segment of the right ICA for protection. Superselective catheterization of the fistula was performed using an Echelon 10 microcatheter, which was advanced into the fistula. Embolization was achieved with detachable coils. Post-procedure DSA confirmed complete obliteration of the CCF **(E,F)**. At discharge, chemosis had regressed, and at the six-month follow-up, both exophthalmos and tinnitus had resolved.

**Figure 3:**
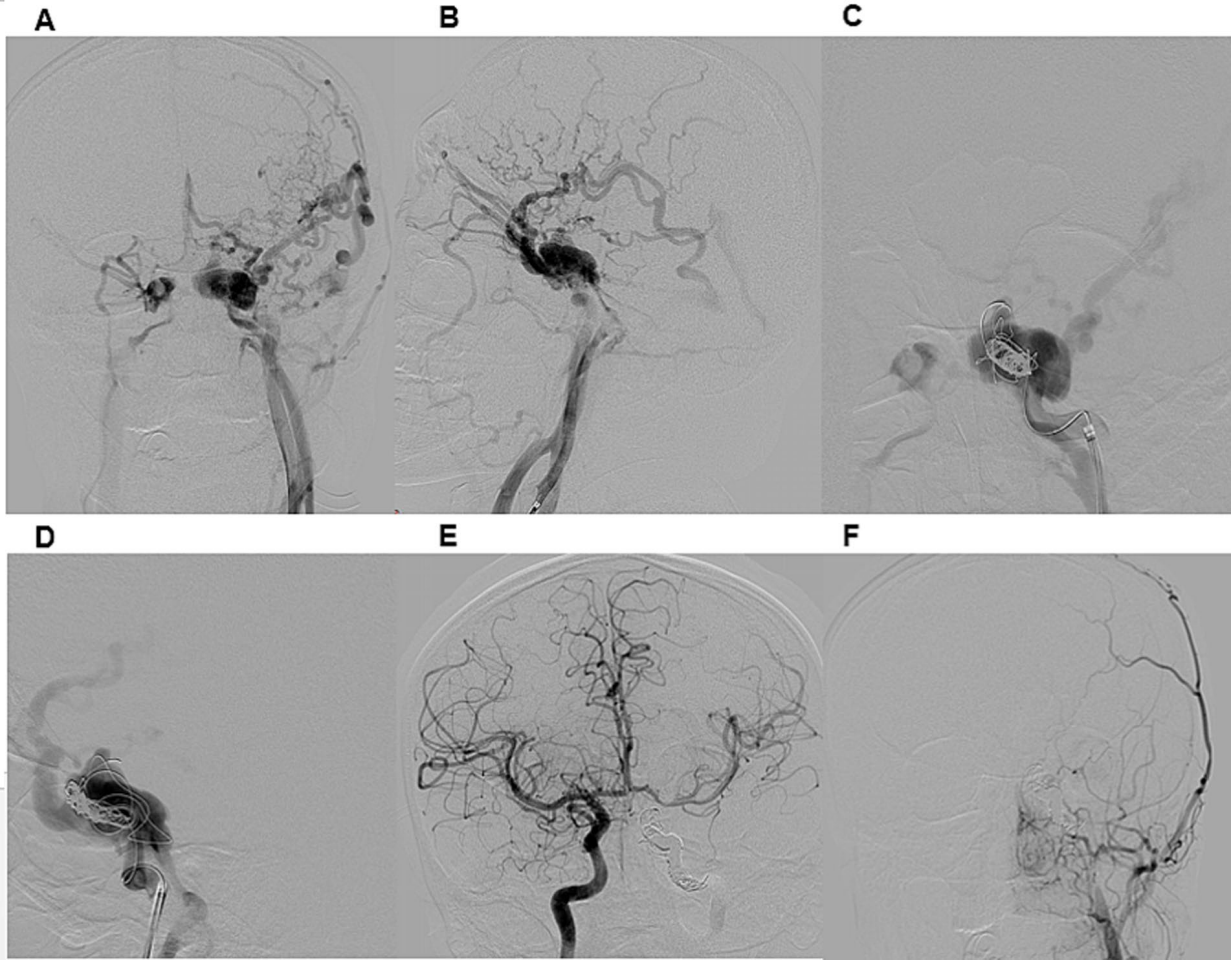
Illustrative case 2. A 21-year-old male presented with a seven-month history of conjunctival injection in the left eye, exophthalmos, and strabismus following a car accident. DSA **(A,B)** confirmed a direct CCF on the left side, classified as Type A according to Barrow et al. and Type 5 according to the Thomas classification system. Venous drainage was retrograde through an enlarged left superior ophthalmic vein, deep and superficial cortical veins, and the intracavernous sinus. Distal ICA flow was reduced, while both the anterior and posterior communicating artery were patent. A right ICA angiogram with compression (Matas maneuver) of the left CCA demonstrated sufficient collateral flow through the anterior communicating artery. The patient underwent transarterial embolization of the CCF and left ICA occlusion under general anesthesia **(C,D)**. A 6F guiding catheter was introduced, and a Headway 17 microcatheter was advanced into the CCF. Embolization of both the CCF and ICA was performed using detachable coils. Post-procedural DSA confirmed complete occlusion of the left ICA and CCF **(E,F)**. At discharge, chemosis had regressed, and at the three-month follow-up, exophthalmos and sixth nerve palsy had also resolved.

The most performed procedure was CCF occlusion via the ICA (Figure 2). Additional techniques included combined occlusion of the CCF and ICA (Figure 3), flow diverter-assisted CCF occlusion, and CCF occlusion via the superior ophthalmic vein (SOV) approach and the inferior petrosal sinus (IPS) approach.

We assessed both the immediate and long-term efficacy and safety of various endovascular techniques and embolic materials.

### 2.3 Follow-up

The primary goal of CCF treatment was to occlude the pathological connections between the carotid artery and its branches and the cavernous sinus while preserving ICA patency. This aimed to reduce the risk of hemorrhage and improve clinical symptoms.

Clinical follow-up was conducted using a standardized 12-question survey administered via telephone interviews. Patients were assessed for: Ocular manifestations: persistence or resolution of vision loss, ocular pain, chemosis, and exophthalmos; Cavernous manifestations: Function of eye movement nerves (presence or absence of diplopia); Cortical manifestations: Occurrence of intracranial hemorrhage, seizures, or focal neurological deficits; Petrosal manifestations: Presence of tinnitus.

Ocular and cavernous sinus symptoms were evaluated by a neuro-ophthalmologist and neurosurgeons during follow-up visits, using comprehensive clinical examinations. Clinical outcomes were classified as follows: Positive outcome: Complete resolution of all symptoms; Moderate improvement: Partial resolution, which includes vision impairment; Negative outcome: No significant improvement in symptoms or the development of additional symptoms, including stroke.

Radiological outcomes were assessed by neurosurgeons and neuroradiologists. Complete obliteration of the CCF was defined as the absence of fistula filling on follow-up angiography performed after embolization. Long-term outcomes were evaluated based on follow-up radiological and clinical assessments.

In cases where recanalization was suspected on MRI during follow-up, diagnostic angiography was performed to confirm the findings. If recanalization was confirmed, a repeat endovascular procedure was carried out.

### 2.4 Statistical analysis

All statistical analyses were conducted using Stata version 18 MP (StataCorp LLC, College Station, TX, USA). Descriptive statistics were used to summarize demographic and clinical characteristics. Continuous variables were reported as means with standard deviations (mean ± SD), while categorical variables were presented as frequencies and percentages (*N*, %).

The Shapiro–Wilk test was applied to assess the normality of continuous variables. For comparisons across the three clinical outcome groups (“No improvement,” “Partial resolution,” “Resolution”), the Kruskal-Wallis test was used for non-normally distributed continuous variables, and Chi-square or Fisher’s exact test was used for categorical variables, as appropriate. Fisher’s exact test was applied when expected cell counts were <5.

## 3 Results

A total of 71 patients were included in the study (Table 1). The mean age was 35.7 ± 12.9 years, with no significant age difference across outcome groups (*p* = 0.97). Most patients were male (71.8%), and sex distribution did not differ significantly by outcome (*p* = 0.57). Presenting symptoms varied, with cavernous+ocular (38.0%) and ocular (25.4%) patterns being most frequent.

**Table 1:** Demographic and clinical characteristics of study participants, *N* (%).

Variable	All *N* (%)	Outcome			*p*-value
		No improvement	Partial resolution	Resolution	
All patients	71 (100)	3 (4.2)	11 (15.5)	57 (80.3)	
Follow-up in months (Mean±SD)	110.2 ± 58.4	130.7 ± 28.5	120.1 ± 59.7	107.2 ± 59.6	
Age (Mean±SD)	35.7 ± 12.9	34.0 ± 14.8	34.9 ± 11.8	35.9 ± 13.2	
Sex					
Female	20 (28.1)	0	4 (20.0)	16 (80.0)	0.571
Male	51 (71.8)	3 (5.9)	7 (13.7)	41 (80.4)	
Presenting symptom					
Cavernous	6 (8.5)	1 (16.7)	0	5 (83.3)	0.302
Cavernous+Ocular	27 (38.0)	2 (7.4)	7 (25.9)	18 (66.7)	
Cavernous+Ocular+Petrosal	10 (14.1)	0	1 (10.0)	9 (90.0)	
Ocular	18 (25.4)	0	1 (5.7)	17 (94.4)	
Ocular+Petrosal	10 (14.1)	0	2 (20.0)	8 (80.0)	
Side					
Left	35 (49.3)	3 (8.6)	7 (20.0)	25 (71.4)	0.095
Right	36 (50.7)	0	4 (11.1)	32 (88.9)	

Out of 71 patients, most CCFs were traumatic in origin (60 cases, 84.5%), while 11 cases (15.5%) occurred spontaneously. Direct fistulas were seen in 62 patients (87.3%). According to the Barrow classification, type A was identified in 62 patients (87.3%), type D in 7 patients (9.9%), type B in 1 patient (1.4%), and type C in 1 patient (1.4%). Anatomical characteristics presented in the Table 2.

**Table 2:** Anatomical characteristics of study participants, *N* (%).

Variable	All *N* (%)	Outcome			*p*-value
		No improvement	Partial resolution	Resolution	
Etiology					
Spontaneous	11 (15.5)	0	2 (18.2)	9 (81.8)	1.000
Traumatic	60 (84.5)	3 (5.0)	9 (15.0)	48 (80.0)	
Anatomical					
Direct	62 (87.3)	3 (4.8)	10 (16.1)	49 (79.0)	1.000
Indirect	9 (12.7)	0	1 (11.1)	8 (88.9)	
Barrow classification					
A	62 (87.3)	3 (4.8)	10 (16.1)	49 (79.0)	1.000
B	1 (1.4)	0	0	1 (100.0)	
C	1 (1.4)	0	0	1 (100.0)	
D	7 (9.7)	0	1 (14.3)	6 (85.7)	
Hemodynamic					
High	62 (87.3)	3 (4.8)	10 (16.1)	49 (79.0)	0.720
Low	9 (12.7)	0	1 (11.1)	8 (88.9)	
Thomas system for classification					
2	3 (4.2)	0	1 (33.3)	2 (66.7)	0.879
3	3 (4.2)	0	0	3 (100.0)	
4	3 (4.2)	0	0	3 (100.0)	
5	62 (87.3)	3 (4.8)	10 (16.1)	49 (79.0)	
Cortical venous reflux					
No	37 (52.1)	1 (2.7)	5 (13.5)	31 (83.8)	0.715
Yes	34 (47.9)	2 (5.9)	6 (17.7)	26 (76.5)	
Contralateral cavernous sinus filling					
No	31 (43.7)	0	3 (9.7)	28 (90.3)	0.168
Yes	40 (56.3)	3 (7.5)	8 (20.0)	29 (72.5)	
Distal ICA flow					
Decreased	27 (38.0)	1 (3.7)	6 (22.2)	20 (74.1)	0.145
No flow	27 (38.0)	2 (7.4)	5 (18.5)	20 (74.1)	
Normal	17 (23.9)	0	0	17 (100.0)	

The most frequently performed surgical procedure was carotid-cavernous fistula occlusion via the internal carotid artery, accounting for 59.2% of cases (Table 3). Additional techniques included combined CCF and ICA occlusion (16.9%), CCF occlusion via the superior ophthalmic vein approach (7.0%), inferior petrosal sinus approach (4.2%), flow diverter-assisted CCF occlusion (4.2%), and middle meningeal artery occlusion (8.4%). We evaluated both the immediate and long-term efficacy and safety of different endovascular approaches (Supplementary Table 1) and embolic materials (Supplementary Table 2).

**Table 3:** Surgical characteristics of study participants, *N* (%).

Variable	All *N* (%)	Outcome			*p*-value
		No improvement	Partial resolution	Resolution	
Surgery details					
CCF occlusion ICA + via IPS	4 (5.6)	0	1 (25.0)	3 (75.0)	0.975
CCF occlusion ICA + via SOV	1 (1.4)	0	0	1 (100.0)	
CCF occlusion via ICA	45 (63.4)	2 (4.4)	7 (15.6)	36 (80.0)	
CCF occlusion+ ICA occlusion	15 (21.1)	1 (6.7)	2 (13.3)	12 (80.0)	
MMA occlusion+ another branch	6 (8.5)	0	1 (16.7)	5 (83.3)	
Number of interventions					
1	57 (80.3)	2 (3.5)	7 (12.3)	48 (84.2)	0.838
2	14 (19.7)	1 (7.1)	2 (14.3)	11 (78.6)	
Treatment approach					
1. transarterial+transvenous 2. transvenous	1 (1.4)	0	0	1 (100.0)	0.529
1. transarterial 2. transarterial	8 (11.3)	1 (12.5)	0	7 (87.5)	
1. transarterial 2. transvenous	5 (7.04)	0	2 (40.0)	3 (60.0)	
transarterial	52 (73.2)	2 (3.9)	8 (15.4)	42 (80.8)	
transarterial+transvenous	1 (1.4)	0	0	1 (100.0)	
transvenous	5 (5.6)	0	1 (25.0)	3 (75.0)	
Embolic material					
Flow diverter+Onyx	1 (1.4)	0	0	1 (100.0)	0.778
Flow diverter+Coils	2 (2.8)	0	0	2 (100.0)	
Onyx	9 (12.7)	0	1 (11.1)	8 (88.9)	
Coils	42 (59.2)	1 (2.4)	7 (16.7)	34 (80.9)	
Coils+Onyx	17 (23.9)	2 (11.8)	3 (17.7)	12 (70.6)	

Out of 71 patients, 57 (80.3%) underwent a single intervention, while 14 (19.7%) required two interventions, indicating the necessity for staged interventions in complex cases. Among those who had one intervention, 84.2% achieved complete resolution, 12.3% had partial resolution, and 3.5% showed no improvement. Similarly, among patients with two interventions, 78.6% achieved complete resolution, 14.3% had partial resolution, and 7.1% showed no improvement.

Imaging follow-up was available for all 71 patients. Complete occlusion was confirmed in 65 of 71 patients (91.5%) on MRI performed at the 6-month follow-up. The remaining 6 patients (8.5%) showed signs of persistent CCF filling on MRI and subsequently underwent digital subtraction DSA for further evaluation (Table 4). The timing of MRI follow-up was uniformly 6 months for all patients. Clinical follow-up was available for a mean duration of 110.18 months (range: 6–193 months).

**Table 4:** Clinical and radiological outcomes, *n* (%).

Clinical outcome	Timepoint	Total patients (*n*)	Recanalization on MRI at 6 Months	
			No (*n*, %)	Yes (*n*, %)
Resolution	At discharge	20	20 (100.0%)	0 (0.0%)
	At 6-month follow-up	57	53 (93.0%)	4 (7.0%)
Partial resolution	At discharge	41	37 (90.2%)	4 (9.8%)
	At 6-month follow-up	11	10 (90.9%)	1 (9.1%)
Unchanged	At discharge	10	8 (80.0%)	2 (20.0%)
	At 6-month follow-up	3	2 (66.7%)	1 (33.3%)
Total		71	65 (91.5%)	6 (8.5%)

Clinical outcomes at discharge showed complete resolution in 20 patients (28.2%), partial resolution in 41 (57.7%), and no change in 10 (14.1%). Notably, all patients with complete resolution had no signs of recanalization at follow-up MRI. At the 6-month follow-up, clinical outcomes had improved, with complete resolution in 57 patients (80.3%), partial resolution in 11 (15.5%), and unchanged status in 3 (4.2%).

No significant associations were found between presenting symptoms, side of lesion, etiology, anatomical type, or Barrow classification and clinical outcome. Similarly, no associations were observed for hemodynamic profile, Thomas classification, cortical venous reflux, or contralateral cavernous sinus filling.

Among treatment-related variables, the occlusion status after the first surgery showed a strong and statistically significant association with clinical outcome (*p* < 0.001), with 96% of patients with complete occlusion achieving full resolution. Other factors such as distal ICA flow (*p* = 0.145), number of interventions (*p* = 0.838), treatment approach (*p* = 0.529), embolic material used (*p* = 0.778) were not significantly associated with clinical outcomes.

Periprocedural complications were observed in 3 cases (4.2%), which included: Coil migration with Solitaire extraction (1.4%), Petrosal ICA dissection (1.4%), and Onyx migration into the middle cerebral artery with Solitaire thromboextraction (1.4%). Despite these complications, no mortality was recorded. One case showed significant temporary worsening of cavernous symptoms (increased chemosis and exophthalmos) in a patient with an indirect CCF (Barrow type D) treated with transvenous embolization using a combination of coils and liquid embolic agents via the superior ophthalmic vein. The clinical worsening occurred on the third day post-procedure, and on the same day, a second transvenous coil embolization via the inferior petrosal sinus was performed, leading to symptom improvement.

## 4 Discussion

In this retrospective, single-center cross-sectional study, we assessed the periprocedural characteristics of endovascular treatment for CCFs, with particular focus on clinical outcomes. According to existing literature, spontaneous closure of direct high-flow CCFs is exceedingly rare, with an estimated incidence of approximately 0.05% of cases (11, 12). Consequently, active therapeutic intervention is generally warranted to prevent serious complications, including progressive visual loss and intracerebral hemorrhage (13, 14).

The decision to treat a dural CCF requires a careful assessment of the risks and benefits, balancing the severity of symptoms against the potential complications of intervention. The initial endovascular strategy is primarily determined by a detailed angiographic analysis of the fistula, particularly the arterial feeders and venous drainage routes (15). This evaluation often involves selective catheterization of the internal and external carotid arteries, along with a meticulous examination of the venous phase, to achieve a comprehensive understanding of the lesion’s angioarchitecture.

Historically, management options for direct CCFs were limited to either conservative observation or surgical trapping, such as ligation of the cervical or intracranial segments of the ICA or occlusion of the CCA (16). These approaches were associated with considerable risk, particularly ischemic complications resulting from compromised cerebral perfusion (17). The evolution of endovascular techniques has markedly transformed the management of CCFs. Open surgical procedures are now rarely indicated and are primarily employed as adjunctive strategies in combination with endovascular interventions, for instance, creating access routes via the transorbital approach (18, 19).

The choice of an optimal treatment strategy depends on several important factors, including the arterial supply, venous drainage pattern, flow dynamics of the fistula, and the patency of the circle of Willis (20). Among the various techniques available, the transarterial approach via the ICA remains the most frequently used (10). Over time, transarterial embolization with detachable platinum coils has proven to be highly effective, achieving both complete angiographic occlusion and favorable clinical outcomes (21). Consequently, it is now regarded as the first-line treatment for direct CCFs, offering a versatile and reliable therapeutic option (22). In most cases, balloon-assisted coiling of the cavernous sinus is routinely used to improve precision and control during coil deployment (23). Coils offer several advantages, including adaptability to different anatomical configurations and the ability to be repositioned during deployment if initial placement is suboptimal (24). However, challenges remain, such as the risk of coil migration and incomplete embolization, especially in cases with compartmentalized cavernous sinuses that require dense packing (25, 26).

In our cohort, coils were the sole embolic agent in 42 patients, predominantly those with traumatic CCFs. In 17 additional cases, coils were combined with Onyx to improve penetration and packing density. The rationale for this combined approach was based on the fistula’s complex morphology and evidence suggesting enhanced occlusion rates (27, 28). In our series, only one case of coil migration was observed. The dislocated coil was successfully retrieved using a stent retriever, and the patient experienced no neurological deterioration following the complication.

Several studies have reported favorable outcomes with the use of covered or flow-diverting stents for the treatment of CCFs via the internal carotid artery, often demonstrating high success rates (29–31). However, these procedures remain technically demanding, particularly in high-flow fistulas, where overlapping arterial and venous phases can hinder accurate navigation and precise stent deployment.

A major concern associated with covered stents is the risk of complications, including endoleaks, acute or delayed in-stent thrombosis, and in-stent stenosis (4, 32). While acute thrombosis is relatively rare, it has been documented in patients undergoing treatment for direct CCFs. Another important limitation is the high cost of covered stents, which substantially increases the overall cost of the procedure (17). This financial burden, combined with the technical challenges and risk of serious complications, limits the routine use of covered or flow-diverting stents in clinical practice (21). In our study, this approach was applied in only three patients, where flow-diverter stents were used in combination with coils and Onyx, depending on the individual angioarchitecture, without any complications.

Onyx embolization for CCFs was first introduced in 2004 (33), and subsequent studies have demonstrated excellent clinical and angiographic outcomes (34, 35). In our experience, Onyx was used as the sole embolic agent in nine patients with low-flow fistulas, while it was combined with detachable coils in 17 cases and with flow diversion in one case. Onyx offers several advantages as a liquid embolic agent: its nonadhesive nature minimizes the risk of microcatheter retention, allowing for prolonged injections and deeper penetration into the cavernous sinus, draining veins, and distal arterial feeders, areas that may be difficult to access with conventional coil embolization (36). Additionally, its controlled precipitation enables intermittent injections with periodic angiographic assessment for precise fistula occlusion (37). In our series, only one complication was observed, which is a migration of Onyx into the middle cerebral artery. The embolic material was successfully retrieved using a Solitaire stent retriever, and the patient experienced no neurological deterioration following the event.

Although transvenous embolization is commonly favored for indirect CCFs due to the relatively straightforward venous anatomy and high occlusion rates up to 94% in some studies (38), it carries a notable risk of complications, which may vary by center and reach up to 20% (39–41). Reported adverse events include intracerebral hemorrhage, ischemic stroke, cranial nerve palsies, trigeminal neuropathy, brainstem infarction, elevated intraocular pressure, and orbital hemorrhage, particularly when access is obtained via the superior or inferior ophthalmic veins (41). Rare complications have also been described, such as the syndrome of inappropriate antidiuretic hormone secretion (SIADH) (42). Additionally, bradycardia and asystole during Onyx embolization of CCFs have been documented (43), possibly due to direct stimulation of the trigeminal nerve, triggering a vagal response via the trigeminocardiac reflex during the transvenous approach.

Despite its effectiveness, the risk profile associated with transvenous embolization has led our center to favor the transarterial approach as the first-line treatment in most cases, particularly when the vascular anatomy permits safe arterial access. In patients with high-flow shunts or complex venous drainage patterns, a combined transarterial and transvenous embolization strategy may be required to achieve complete and durable occlusion (26). In our series, seven patients required this combined approach. One patient underwent a simultaneous procedure via the transfemoral artery and the inferior petrosal sinus, while six patients were treated in a staged manner, with transarterial embolization performed first, followed by transvenous embolization. Notably, no intraoperative complications, including those previously described, were observed in these cases.

ICA sacrifice in combination with CCF occlusion is a viable treatment option, particularly for patients with direct-type CCFs who demonstrate adequate collateral circulation during balloon test occlusion (BTO). During BTO, arterial pressure is typically reduced by approximately 20% to evaluate the adequacy of cerebral perfusion via the circle of Willis.

ICA sacrifice is generally reserved for cases involving complete ICA transection or a large fistulous defect that precludes successful cavernous sinus occlusion while preserving the parent artery. The need for ICA sacrifice should be considered in all patients with direct CCFs. A suspected ICA transection is typically indicated by failure to visualize the distal ICA on initial angiography; however, this finding may also result from high-flow shunting through the fistula (44). Persistent non-visualization of the distal ICA following partial coiling of the cavernous sinus strongly supports the diagnosis of complete transection, warranting occlusion of both the ICA and the cavernous sinus (45, 46).

In our series, the strategy of identifying ICA transection and proceeding with combined ICA and cavernous sinus occlusion was employed in 15 patients. Although the underlying indications varied, early evaluation of ICA integrity provided the flexibility to adapt the treatment plan accordingly.

At our institution, all vascular neurosurgeons are dual-trained and capable of performing emergency superficial temporal artery to middle cerebral artery (STA-MCA) bypasses. This surgical expertise is critical in cases where a false-negative BTO could lead to ischemic complications. Consequently, ICA sacrifice is a treatment option best suited for centers equipped with neurosurgeons experienced in microsurgical revascularization techniques.

According to the literature, endovascular treatment of carotid-cavernous fistulas (CCFs), irrespective of the specific technique or vascular access route employed, results in clinical improvement in approximately 60 to 95% of cases (17, 38, 47, 48). Ophthalmic symptoms such as proptosis, chemosis, and pulsatile tinnitus often resolve rapidly following embolization. In contrast, cranial nerve palsies and visual deficits tend to exhibit a more protracted course of recovery, frequently requiring several months to achieve full resolution (49).

In our cohort, among patients who achieved complete occlusion following the initial endovascular intervention, 96% experienced complete clinical resolution, while 4% demonstrated partial improvement. These findings suggest that early therapeutic intervention may play a critical role in the recovery of ocular symptoms and visual function. This hypothesis is further supported by a recent systematic review and meta-analysis conducted by Ali Al-Shalchy, which underscored the significance of timely treatment in optimizing visual outcomes in patients with CCF (50).

In total, complete symptom resolution was achieved in 57 patients (80.3%), while 11 patients (15.5%) demonstrated partial clinical improvement. These outcomes are consistent with findings from prior systematic reviews and meta-analyses, which have shown that clinical improvement following endovascular treatment of carotid-cavernous fistulas is not significantly influenced by the choice of embolic material, vascular access route, or the underlying anatomical or etiological classification (10, 51). Similarly, our data indicate that complete clinical recovery was strongly correlated with successful angiographic obliteration of the fistula during the initial intervention (20).

Importantly, no cases of procedure-related mortality or stroke were observed during the follow-up period, underscoring the safety and efficacy of endovascular treatment in this patient population.

Although this study provides important insights into the endovascular treatment of CCFs, there are a few limitations to consider. First, its retrospective, single-center design may limit the generalizability of the findings. Second, the 16-year study period spans significant advancements in endovascular techniques, embolic materials, and operator experience, which may have introduced heterogeneity in treatment approaches and outcomes, complicating direct comparisons. Finally, a major limitation of our study is the limited availability of long-term DSA follow-up. This may have led to underdetection of small residual or recurrent fistulas, which could contribute to late recurrences. Future prospective, multicenter studies with standardized outcome measures are needed to validate these results and optimize treatment strategies.

## 5 Conclusion

Endovascular therapy is the treatment of choice for carotid-cavernous fistulas, offering high occlusion rates with a low risk of complications. Both transarterial and transvenous approaches demonstrate comparable efficacy, with no significant differences in clinical outcomes. These findings support the use of endovascular embolization as the first-line therapeutic strategy, with treatment individualized according to each patient’s vascular anatomy. Achieving complete occlusion significantly reduces the risk of serious complications, including visual impairment and ischemic events. Moreover, early intervention appears to play a key role in optimizing clinical outcomes.

## Data Availability

The original contributions presented in the study are included in the article/Supplementary material, further inquiries can be directed to the corresponding author.
