# Supplementary material for: Endovascular management of carotid-cavernous fistulas: a 16-year retrospective analysis of multimodal treatment strategies and long-term clinical outcomes
Source: Front Neurol. 2025 Aug 26;16:1625899. doi: 10.3389/fneur.2025.1625899 (PMC12417146; doi:10.3389/fneur.2025.1625899)
Supplement: Supplementary file 1 [file Table_1.docx]

Supplementary Tables

***Supplementary Table 1. Endovascular treatment strategies for direct and indirect Carotid-Cavernous Fistulas***

|  | | ***Treatment approach*** | | | | | | Total | |
| --- | --- | --- | --- | --- | --- | --- | --- | --- | --- |
|  |  | 1. transarterial+transvenous 2.transvenous | 1.transarterial 2. transarterial | 1.transarterial 2. transvenous | transarterial | transarterial+transvenous | transvenous |  |  |
| ***Type of CCF*** | ***direct*** | 0 | 7 | 2 | 50 | 1 | 2 | 62 |  |
|  | ***indirect*** | 1 | 1 | 3 | 2 | 0 | 2 | 9 |  |
| ***Total*** | | 1 | 8 | 5 | 52 | 1 | 4 | 71 |  |

***Supplementary Table 2. Types of embolization materials used in transarterial and transvenous embolization***

| ***Embolic material*** | | | | | | | |
| --- | --- | --- | --- | --- | --- | --- | --- |
|  | | Coils | Coils + Onyx | FD+Coils | FD+Onyx | Onyx | Total |
| ***Treatment approach*** | 1. transarterial+transvenous  2.transvenous | 0 | 1 | 0 | 0 | 0 | 1 |
|  | 1.transarterial  2. transarterial | 5 | 1 | 1 | 1 | 0 | 8 |
|  | 1.transarterial  2. transvenous | 0 | 3 | 0 | 0 | 2 | 5 |
|  | transarterial | 36 | 9 | 1 | 0 | 6 | 52 |
|  | transarterial+transvenous | 0 | 1 | 0 | 0 | 0 | 1 |
|  | transvenous | 1 | 2 | 0 | 0 | 1 | 4 |
| **Total** | | 42 | 17 | 2 | 1 | 9 | 71 |
